# Virtual Teams: Taking Stock and Moving Forward

**DOI:** 10.1177/10464964241274129

**Published:** 2024-08-26

**Authors:** Lisa Handke, Patrícia Costa, Thomas A. O’Neill

**Affiliations:** 1 Friedrich-Alexander-Universität Erlangen-Nürnberg, Germany; 2 Instituto Universitário de Lisboa (ISCTE-IUL), BRU-IUL, Portugal; 3 University of Calgary, Canada

**Keywords:** virtual groups, teamwork, computer-mediated communication

## Abstract

The papers in this Special Issue show that virtual teamwork is a complex phenomenon that depends on a multiplicity of team, task, and environmental factors. In this editorial, we begin with a short review of the main perspectives through which virtual teams have been studied. From there, we move to an overview of the papers in this Special Issue. To conclude, we discuss potential avenues for future research based on the collection of papers in this issue.

Twenty-four years into the new millennium, the world we live in has changed and evolved in ways most science fiction novels or movies did not fully anticipate. While we do not (yet) use technology for time travel, teleportation, or flying cars, we are undoubtedly extremely reliant on technology to communicate, coordinate, and collaborate with others. In 2000, only about half of American homes had broadband internet, whereas over 90% have internet access today (US Census Bureau, 2022). This broadband expansion is also reflected globally, with internet access skyrocketing from less than 7% of the global population in 2000 to two thirds of the global population today (Hillyer, 2020; Kemp, 2024). In a similar vein, the use of mobile devices has exploded, and there are now as many mobile subscriptions in the world as there are people (Ericsson, 2024).

At work, technology has established itself as an irreplaceable asset to collaboration, giving rise to teams that are commonly called virtual—to a greater or lesser extent. Virtual teams are teams that primarily collaborate using information and communication technologies, and whose members sometimes are—but do not have to be—geographically dispersed (e.g., Gilson et al., 2015; Raghuram et al., 2019). Ongoing changes of work, the increased ease of mobility, ubiquitous presence of communication technologies, and the Covid-19 pandemic have fueled the possibility and necessity of virtual teamwork. Yet, even in the post-pandemic world, organizations and workers alike are still grappling with the reality of collaborative forms of remote and hybrid work. The aim of this Special Issue is to take stock of what we know about virtual teams that will remain true and important, as well as to move forward and examine new perspectives on virtual teamwork in post-pandemic times.

We begin this Editorial with a short review of the main perspectives through which virtual teams have been studied. From there, we move to an overview of the papers in this Special Issue. To conclude, we discuss potential avenues for future research based on the collection of papers in this issue.

## Taking Stock

Our knowledge of virtual teams stems from decades of research, spurred by both the evolution of technology and by different disciplines such as communication, psychology, management, and information systems (Gibbs et al., 2017; Raghuram et al., 2019). What we know so far about teams that collaborate using information and communication technology has its roots in the literature in telecommuting (e.g., Gajendran & Harrison, 2007; Kossek et al., 2006), computer-mediated work (e.g., Daft et al., 1987; McFarland & Ployhart, 2015), mainstream small groups and teamwork literature (e.g., Hackman, 1987; Steiner, 1972), and the virtual/distributed teams literature (e.g., Gibson & Gibbs, 2006; Hill & Bartol, 2016).

Describing a complete picture of the accumulated knowledge on virtual teams is beyond the scope of this Editorial and has been done elsewhere (e.g., Gilson et al., 2015; Raghuram et al., 2019). Overall, these works tend to focus either on geographic dispersion and technology dependance, exploring how these aspects influence individual attitudes and outcomes, team dynamics, or task-related outcomes. For example, there is a considerable amount of work that highlights the importance of task-technology fit for the effectiveness of information exchange, or the importance of establishing norms and expectations around technology usage. Also, a vast amount of research has explored how virtuality influences conflict, trust, or psychological safety of team members, together with an exploration of the role of leadership for team effectiveness. The results of these works, however, have not always been univocal (Handke et al., 2021; Purvanova & Kenda, 2022), such that a further stream of research has concentrated on subjective experiences of virtuality, next to the more structural or objective features of technology use or geographic dispersion (e.g., Handke et al., 2021; Watson-Manheim et al., 2012). Hence, recent work has started to explore the collective perceptions of virtuality in teams, and their effect on collaborative work (e.g., Costa et al., 2024; Handke et al., 2024).

## Special Issue Contributions

We believe the papers included in this Special Issue have responded well to the aim of our call, particularly in terms of moving beyond traditional correlational and cross-sectional designs. First, the paper by Driskell et al. (2023) conceptually explores the fluidity in virtual teams, that is, the rapid assembly of members with limited prior experience in working together to perform immediate and time-sensitive tasks (e.g., control teams). Merging the concept of team virtuality with the concept of fluidity, the authors describe four main types of teams, defined by their (high or low) degree of virtuality and (high or low) degree of fluidity. They discuss the impact of each for team development, team processes and emergent states, and propose strategies to promote the effectiveness of virtual and fluid teams.

Second, Hoffmann et al. (2023) consider blink synchronization—an operationalization of physiological interpersonal synchrony defined by the authors as “the temporal coordination of physiological processes between two or more individuals”—to be a proxy for a shared mental model development among virtual team members. The authors argue and review research suggesting that eye gaze can serve as a cue that directs others’ attention to specific objects or can help follow other person’s thought processes, and that the coupling of eye movements (i.e., blink rate synchronization) thus reflects how well interaction partners achieve a shared mental awareness about an object or mental representation. In an experimental design featuring dual eye tracking devices, and in support of this assumption, blink synchronization was shown to be associated with effective problem solving in dyads, particularly over time. The authors discuss implications for real-world virtual teams, including the potential of including technology-based methods of data collection and the need to better understand how to enhance behavioral synchronization to increase team performance.

Third, in their paper on interdependence in virtual teams, Kanse et al. (2023) experimentally explore the impact of process and resource interdependence on both task and creative performance in virtual teams. Process interdependence (i.e., team members’ interconnectedness regarding workflow) was found to be beneficial for both types of performance, whereas resource interdependence (i.e., team members’ interconnectedness regarding access to critical resources) decreased creative performance. The results of this study underline the importance of clearly distinguishing between different dimensions of interdependence and offer both theoretical implications in terms of unraveling the mixed effects of interdependence found in prior research on virtual teams (see Handke et al., 2020), as well as practical implications when it comes to designing optimal conditions for virtual teamwork.

Fourth, Grabowski et al. (2024) develop a conceptual framework to guide research on immersive extended reality (XR) group meetings. With the merit of bringing together meeting science, human-computer interaction, and group dynamics literature, this paper sets the stage for future progress in building effective immersive XR environments that enhance and facilitate team collaboration. The authors also offer a pilot study based on their framework, which finds that even without prior XR experience, participants experienced satisfying immersive meetings under certain conditions. Moreover, participants quickly habituated to using the technology. Even though these empirical findings remain preliminary, this paper offers a range of opportunities for more interdisciplinary research on the future of workplace meetings.

Fifth, the conceptual work of Handke et al. (2024) focusses on the nature and effects of hybrid work (i.e., any combination of one’s work time spent across organizational and other—typically domestic—work settings) at the team level. The authors critically analyze how the extant literature on virtual teams (focusing on geographic dispersion, on technology usage, and on subjective perceptions of virtuality) can be leveraged to inform our understanding of hybrid teamwork. Based on both existing knowledge and its limitations within the extant literature, the authors conclude this paper by mapping out pressing questions to guide future research on hybrid teamwork. Researchers interested in hybrid teams will find many interesting theoretical questions to pursue.

## Moving Forward

Based on the contributions in this Special Issue, we highlight two possible pathways to advance research on virtual teams. The first pathway concentrates on how we conceptualize and operationalize virtual teamwork. The heterogenous findings linked to team virtuality (e.g., Carter et al., 2019; De Guinea et al., 2012; Purvanova & Kenda, 2022) suggest that current conceptualizations and measurement approaches fall short in capturing the actual experience of virtual teamwork. Accordingly, rather than measuring the extent of virtual teamwork through geographic dispersion and technology dependence, some scholars have recently begun to concentrate on the subjective experience of working virtually (e.g., Handke et al., 2021; 2024). Future research could extend the nomological network around these subjective experiences through exploring potential antecedent and outcome factors. At the same time, future research could also look at alternative ways of capturing structural properties such as geographic dispersion or technology dependance at the team level. Much of extant research has used compositional approaches to capture virtual teamwork (i.e., team average scores of e.g., face-to-face interaction, Maynard et al., 2012, technology reliance, Brown et al., 2020), assuming considerable agreement among team members. Some scholars have used indices that capture geographic configuration (e.g., Ganesh & Gupta, 2010; Hoch & Kozlowski, 2014, see also O’Leary & Cummings, 2007), which acknowledge that there may be within-team differences in co-location. However, as discussed in Handke et al. (2024) contribution in this issue, we currently lack research that captures the reality of virtual teamwork as it is enacted today, namely often as a result of team members who engage in hybrid work. In practice, this means not only that there will be within-team differences in geographic dispersion and technology dependance but also that these differences will constantly change. We thus urgently need research that captures both the nature and the effects of these dynamics.

The second pathway focusses on the information and communication technologies that are central to virtual teamwork. The ongoing and fast-paced technological developments we observe can both impact how virtual teamwork is enacted and experienced and also contribute to innovative research designs. Virtual reality spaces of all kinds need to be explored in terms of how they shape and constrain virtual work, from choosing one’s avatar to the degree of immersion these spaces provide. Tracking technologies such as wearables can allow for longitudinal designs and the collection of biometric data, going beyond more traditional self-report cross sectional designs. Including robots and artificial intelligence agents as part of the team may help us build knowledge on human collaboration, thereby suggesting that we should capitalize on the new work happening in human-autonomy teams (O’Neill et al., 2022). O’Neill et al. (2023) also argued that human-autonomy teams are by their very nature virtual—calling for an integration of work in the virtual team and HAT arenas. In a similar vein, opening the dialogue between disciplines that tend to develop research separately (e.g., organizational behavior and computer science) will likely enrich research designs and findings, underscoring the breadth of areas in teamwork in general, and virtual teamwork in particular.

## Conclusion

The papers in this Special Issue show that virtual teamwork is a complex phenomenon that depends on a multiplicity of team, task, and environmental factors. Specifically, both the nature and effects of virtual teams may change and evolve as a function of their members (e.g., team members’ experience in working together, i.e., team fluidity), the organization of collective taskwork (e.g., interdependence), work practices (e.g., hybrid work), or the technologies teams use to interact with (e.g., the degree of immersion or synchronicity). Given that virtual teamwork is a phenomenon already more or less applicable to most organizational teams—particularly as we move from virtual to hybrid teamwork—we thus believe that this Special Issue offers not only important insights to research and practice around virtual teams but for teamwork more generally (see also Gilson et al., 2021).
